# New Drugs and Preparations

**Published:** 1894-03-24

**Authors:** 


### NEW DRUGS AND PREPARATIONS.

[We shall be glad to receive, at our Office, 428, Strand, London, W.C., from the manufacturers, specimens of all new preparations and drugs which may be brought out from time to time.]

### CAFFEINE.

Keerlein[1], after some ingenious experiments, has found that caffeine in small doses increases by about 18 per cent. the amount of oxygen consumed. On the contrary, caffeol exercises no influence on the consumption of oxygen. Faisans[2] has been struck by the number of cases in which he has seen much cerebral excitement, violent delirium, and insomnia follow the use of caffeine. In one case there were hallucinations, and he suggests that sometimes the hallucination and delirium which are set down as due to some specific disease are really caused by the caffeine which has been administered as a stimulant. This, he thinks, is especially likely when the patients are alcoholic or neurotic. Czarkowski[3] calls attention to the contrary indications against the employment of caffeine in cases of alcoholism. He reports three cases in which intense excitement followed its use, and therefore he thinks that in such patients we should always begin with a small dose, and gradually increase it if necessary. Vergely[4] records two cases of caffeine delirium, in a discussion in connection with which Dr. Faisans said he had heard of several cases of a similar kind, and he thinks that on the whole delirium is tolerably frequent after the administration of caffeine, but he does not regard it as in any way dependent upon the integrity of the kidney.

[1] Pfeúger's Archiv. Bd. LXI., S. 165. [2] Nouv. Rem., May 24, 1893. [3] Vratch, 1893, No. 4, p. 105. [4] La Semaine Médicale, June 16, 1893.

